# Supplementary material for: Are carbon emissions trading and green financial instruments synergistic? -Comprehensive quantitative research based on content analysis
Source: PLoS One. 2024 Mar 7;19(3):e0298601. doi: 10.1371/journal.pone.0298601 (PMC10919601; doi:10.1371/journal.pone.0298601)
Supplement: S1 Data — (ZIP) [file pone.0298601.s001.zip › Data/地级市Z_F工作报告.zip!/░▓╗╒/╨√│╟╩╨/└┤╫╘╣1⁄2╓┌║┼í╛┬φ┐╦╩2╛▌═°í┐_2011.docx]

——2012年1月11日在宣城市

第三届人民代表大会第二次会议上

市人民政府市长	虞爱华

各位代表：

现在，我代表市人民政府，向大会报告政府工作，请予审议，并请市政协委员和其他列席人员提出意见。

一、2011年工作回顾

刚刚过去的2011年，是“十二五”起步之年，也是本届政府开局之年。面对复杂严峻的宏观经济形势，全市人民在市委的坚强领导下，认真贯彻落实党中央、国务院和省委、省政府的各项决策部署，坚持在发展中谋转型、在转型中促发展，在2010年度荣获省政府目标管理考核第二名，五县（市）进入全省二十个科学发展先进县的基础上，全市经济社会继续保持了十分强劲的发展势头，主要经济指标增幅实现“两个超过”：超过年初人代会确定的目标，超过全省平均水平。办成了一批群众期盼多年的大事好事，为“十二五”大发展奠定了更加坚实基础。

经济总量迈上新台阶。全年完成生产总值671.4亿元，增长14.1%，增幅居全省第三位，创历年最好位次。规模工业增加值346.1亿元，相当于2000—2007年八年总量，增长24%，高于全省2.9个百分点，增幅居全省第五位。财政收入118.6亿元，超过2000—2006年七年总量，增长40%，高于全省12.4个百分点，增幅居全省第四位；其中地方财政收入68.4亿元，超过2000—2006年七年总量，增长37.2%，高于全省9.9个百分点；财政收入占生产总值的比重达到17.7%。固定资产投资635.6亿元，超过2000—2006年七年总量，增长30.5%，高于全省2.9个百分点，增幅居全省第六位。社会消费品零售总额228.9亿元，增长17.7%。进出口总额9.95亿美元，超过2000—2006年七年总量，增长43.9 %，高于全省14.8个百分点，总量、增幅分别居全省第六位和第四位；其中,出口8.5亿美元，超过2000—2006年七年总量，增长45.7%，高于全省8.1个百分点，总量居全省第四位。实际到位省外资金1272亿元，超过2000—2009年十年总量，增长63%，其中亿元以上项目到位资金382.5亿元，居全省第三位。实际利用外资3.1亿美元，相当于2000—2006年七年总量，增长48.6%，高于全省17.8个百分点，增幅居全省第六位。城市居民人均可支配收入17995元，增长18.8%，增幅居全省第三位；农民人均纯收入7844元，增长17.9%，总量居全省第六位。

实现了三大愿望。期盼几十年的水阳江下游“开卡”工程获得国家发改委批准，并正式动工。宣城设立海关、出入境检验检疫局已经中央相关部门正式批准。合工大宣城校区获教育部正式批准并开工建设，不仅圆了宣城人民二十多年来的本科院校梦，也使我市成为继合肥之后在全省第二个拥有“211工程”大学的城市。

一年来，我们主要做了以下六个方面工作：

（一）坚持不懈调整结构，产业转型明显加快。工业优化升级持续推进。工业化率达到43.9%，较上年提高5.5个百分点。新增规模以上工业企业109家，总数达到689家，居全省第六位。工业经济效益综合指数达到334.7，创历史新高。完成工业投资354.3亿元，增长40.8%，增幅居全省第三位。积极培育战略性新兴产业，实现产值122.6亿元，增长70.2%。中鼎橡胶密封产品项目列入国家战略性新兴产业投资计划，宣州博瑞特高效节能锅炉产业化、旌德煤矸石综合利用等项目列入国家环资项目计划。博迪新材料、精超风光发电设备等项目加快建设。中鼎通用发动机项目已经省发改委备案。高新技术产业发展明显提速。实现总产值280亿元，增加值82亿元，分别增长66%和34%。新增省级创新型企业4家、工程技术研究中心4家、企业技术中心6家，新增高新技术企业23家、高新技术产品121项。新增专利申请量2693项，授权量2285项。乾正光电大功率LED封装、源光电器超高温电容器高技术产业化示范项目通过国家验收。郎溪特种设备制造基地入选省级首批新型工业化产业示范基地，省特检院郎溪检测中心建成使用。新增黄山恒久链、生信铝业、宁国东方碾磨材料等中国驰名商标3个。中鼎密封件公司、中国宣纸集团跻身省“自主创新品牌示范企业”，宣酒集团等4家企业荣获“安徽省质量奖”。传统优势产业稳步提升。生信新型铝型材、广德磐石液动油压、宁国中鼎橡塑、绩溪非标异型链生产研发基地、旌德黄山胶囊等101个技改项目建成投产，鑫鸿交通、宣州华峰重工、广德金鹏等技改项目顺利推进，司尔特70万吨缓释复合肥、徽铝建筑彩铝、泾县泾鼎数控机床二期等266个技改项目开工建设。新能源汽车电机等15个项目列入国家重点产业振兴和技术改造项目计划。宣城海螺水泥二期、国投宣城电厂二期获准开展前期工作。节能减排工作进一步加强，完成了年度目标任务。拆除落后生产线13条，关闭小化工、小造纸、粘土砖生产企业58家。

服务业发展势头强劲。服务业增加值219.4亿元，增长10.7%，其中交通运输业、批发零售业增加值分别增长8.3%和9.4%。全市亿元以上服务业项目42个，总投资额308亿元，其中，市本级重点项目26个，总投资近210亿元。双桥物流园区开工建设，起步区签约项目10个，总投资178亿元。百汇物流和中国茶府项目加快建设，农副和农资产品2个批发市场项目已经开工。宣城世纪联华建成运营。亚夏汽车城、麦莎广场已经竣工。仁和时代广场主体工程封顶。国购广场、星隆国际广场等项目进展顺利。广德大润发超市投入使用，郎溪义乌商贸城开工建设。“万村千乡市场工程”和“新网工程”深入实施。文化产业快速推进。15个项目进入省“861”项目库，8个项目获国家和省专项资金扶持，4个企业跻身省第三批文化产业示范基地。夏渡文化创意产业园近4平方公里起步区开始建设，文房四宝交易中心主体工程即将完工，书画交易中心即将动工建设。中国工艺礼品城、徽文化展示基地等项目已经签约，动漫衍生品交易基地、少儿科普产业基地达成合作意向。广电集团组建进展顺利，市广电文化产业有限公司和改组后的市文房四宝发展有限公司开始运营。泾县桃花潭文化艺术中心一期工程、绩溪徽菜产业文化园建成使用。绩溪上庄徽文化产业示范基地初具规模。中国宣纸集团和胡开文墨业公司入选首批国家级“非遗”生产性保护示范基地。绩溪良才墨业“艺粟斋”获得“中华老字号”称号。旅游业发展继续加快。全市新建、在建旅游项目128个，总投资额412亿元，其中，5亿元以上项目23个。全年国内外游客接待量达到870万人次，增长35.5%；实现旅游总收入61.3亿元，增长39%，占生产总值的9%左右。太极洞被评为全国低碳旅游示范景区。大云岭红色旅游景区项目列入省十大旅游重点工程。敬亭山、金梅岭、徽杭古道通过4A景区验收。绩溪国际、郎溪瀚海、泾县皖南国际、宁国金色王朝等高星级酒店投入使用，宣城伟业酒店、广德木子宾馆、绩溪上河等高星级酒店即将运营，新港生态湖和敬亭湖宾馆、万豪酒店、文苑木子山庄、旌德灵芝大酒店等项目开工建设。郎溪天子湖休闲度假城项目签约。成功举办第五届中国文房四宝文化旅游节、第四届中国徽菜之乡美食文化节、中国（宁国）休闲农业与魅力乡村发展论坛暨农业休闲与乡村旅游系列活动。

现代农业不断升级。宣州洪林现代农业示范区进入省级示范区行列，宁国南山食品产业园成为首批国家农业产业化示范基地。新增2户国家级农业产业化龙头企业，总量达到6户。新增省级农民专业合作示范社10个，位居全省首位。建立水稻核心示范区75万亩、创建高产示范区20万亩。全年粮食总产137.8万吨，增长2.5%。市粮食产业园区5万吨粮食储备库开工建设。年销售额超5亿元家禽企业达到7家，其中超10亿元的5家。农产品加工产值350亿元，增长35%。林业总产值150亿元。油茶面积突破万亩。烟叶种植面积达到7.6万亩。新增国家级标准化畜禽养殖示范小区2个、农业部地理标志认证产品5个。“三品一标”总数占全省十分之一。农业规模经营取得进展，新增流转耕地9.5万亩。宁国、绩溪被认定为全国休闲农业与乡村旅游示范县。恩龙木屋村荣获全国休闲农业与乡村旅游示范点称号，成为全省唯一的全国休闲农业与乡村旅游五星级企业。郎溪成为全省唯一的城乡一体化综合配套改革试点县。

（二）坚定不移招商引资，承接产业转移成效显著。在皖江城市带承接产业转移示范区考核中名列第三。承接平台进一步提升。市开发区强力推进西扩区域“十路一桥”工程建设，“三横三纵”主次干道框架已经拉开，6平方公里的汽车零部件产业园和5平方公里的光电产业园正式启动。郎溪开发区跻身“中国最具发展潜力开发区100强、最佳投资服务环境开发区”行列。宁国开发区获得省创新型园区称号。广德、宁国分别被评为全省优势产业十强县和投资环境十佳县。项目体量进一步扩大。新签亿元以上省外投资项目286个，其中5亿元以上项目45个，10亿元以上项目18个。总投资20亿元的中鼎产业园、22亿元的绩溪高铁铝合金电缆、12亿元的华明飞彩低速汽车和15亿元的蓝宝石衬底材料等项目开工建设。总投资1亿美元的丽车木业新型材料等外资项目签约。合作发展进一步加快。积极参与皖粤经贸对接活动，共签约项目13个，投资总额39.7亿元。大力推进与央企和知名民营企业的合作，对接项目已开工224个，完成投资近300亿元。市开发区一年开工建设项目超百个。郎溪无锡工业园和经都产业基地、广德PCB和汽摩配产业园、宁国医疗器械产业园等园区在建项目350多个。

（三）多方筹资加快基础设施建设，城乡面貌持续改观。城市建设再添亮点。编制完成《宣城市城市近期建设规划》、《城市南部片区概念性总体规划》等。市本级完成城市建设投资110亿元，其中房地产投资48亿元，新开工和续建项目91个。“二环八射”工程取得新进展，环城大道3、4标段和水阳江大道西北段、宣水路一期已经通车，向阳大道1、2标段和宣古路一期、薰化路三期等正在建设。梅溪公园、响山公园和宛溪河整治一期工程对外开放。敬亭山风景区初步完成22项工程。扬子鳄湖（暂定名）核心景区工程、市图书馆、体育中心、规划馆、高速公路东西出入口公园等开工建设。宛溪河整治二期和泥河、道叉河整治项目正在做扎实细致的准备工作。北门旧城改造、宣中新校区等项目顺利推进。“数字城管”平台建成使用，在全省率先建成数字执法平台。通信基础设施建设加快，“光网城市”建设位居全省前列， “无线城市” 基本覆盖全市城区。交通建设再掀高潮。全年完成投资28.9亿元。绩黄高速建成通车，宣宁、芜雁高速路基工程基本完工，合福高铁宣城段、宁绩高速和宣广高速宣城东、西出入口改扩建工程进展加快，宁千高速动工建设，皖赣铁路扩能改造和溧广高速、绩溪至谭家桥高速、205国道宣城段示范工程和宣泾快速通道前期工作扎实推进。鸿旌路梅岭隧道及定十路已经开工，宣狸快速通道、217省道绩溪至长安段和芜申运河宣城段建设加快。水利建设再创佳绩。出台了加快全市水利改革发展的实施意见。城东联圩和溪口枢纽除险加固工程加速推进。41座水库除险加固工程全面竣工，63座水库完成除险加固主体工程。继续开展了青弋江灌区续建配套和宣州东门渡节制闸等3座大中型水闸除险加固前期工作，郎溪中斗闸、绩溪扬溪源水库项目已经省发改委立项。启动了港口湾水库灌区和牛岭水库、城市第二水源等工程前期工作。农村饮水安全工程全面完工，解决了18万人饮水安全问题。面对旱涝急转、连续强降雨袭击的严峻形势，科学调度，成功战胜1999年以来水阳江流域时间最长的一次洪水，全年“虽有洪旱、但无大灾”。县乡村建设再展新貌。全市城镇化率达到45%，比上年提高近2个百分点。宣州区正在推进新型城镇化建设。郎溪大力推进城市“东拓、北扩、南延”，东部新区初具规模。广德实施“四区同城”战略，城市形象显著提升。宁国推进“三城联创”活动，城市品位不断提高。泾县实施城市建设“十大基础工程”，城市功能日益完善。绩溪加快“三区一廊”建设，城市东进步伐加快。旌德城市建设全面提速，新城框架已见雏形。全市扎实开展“美丽和谐乡村”建设，稳步推进村庄整治、危房改造和清洁工程。泾县、绩溪成为省级村庄整治工作试点。生态建设再上台阶。创建省级园林城市通过验收。创建国家生态市和国家环保模范市工作全面启动，宁国、绩溪申报国家级生态县（市）通过环保部验收。新增国家级生态乡镇19个、生态村6个，新增省级生态乡镇8个、生态村15个。

（四）千方百计化解难题，促进经济快速发展。针对发展中遇到的种种难题，逐一深入研究，提出应对之策。为了化解融资难问题，出台了鼓励企业上市政策意见。直接融资总额超过40亿元，达到历史最高水平。司尔特首发上市，江南化工实现定向增发，中鼎股份发行可转债，市国投公司首次获准发行10亿元公司债券。新开业金融机构18家。徽商银行宣城支行成功升格并设立广德支行，交通银行宣城分行正式开业，中行郎溪支行正式挂牌，泾县、旌德成立农商行，在全省率先完成信用联社改革改制任务。宁国民生村镇银行获准筹建。邮储银行在全省市级分行率先开办票据业务。积极开展政银企对接合作，签约项目数、贷款数均超过上年水平。全年银行业金融机构新增贷款再次超百亿元。小额贷款公司累放贷款35亿元。融资性担保机构累计担保贷款40多亿元。为了化解用地难问题，积极争取点供指标和追加计划，整理清收闲置土地，加大用地报批力度，已经累计获得国家和省批准用地面积2.96万亩。将供地指标与项目投资强度、建筑密度和财税贡献率挂钩，大幅度提高土地使用税标准，提高节约集约用地水平。宁国积极开展统筹城乡土地使用制度综合改革试点工作，成立全省首家土地股份合作社。为了化解用电难问题，努力加快电力设施建设，建成2座110千伏变电站，新开工建设和扩建2座220千伏变电站、市中心变等4座110千伏变电站，投产线路近40公里、变电容量176万千伏安。全年完成购网电量 68亿千瓦时，增长17.7%。加快实施农网升级工程，升级改造线路1713公里。为了化解企业经营难问题，在全省率先出台含金量高、操作性强的政策，从鼓励项目建设、减轻企业负担等9个方面加大对企业的支持力度。建立并落实市领导联系重点企业制度，对52户重点企业实行“一企一策”、“一企一组”帮扶措施。组织开展产销对接活动，帮助企业拓展市场。通过建立长期劳务合作基地、组织开展专场招聘活动、加强就业培训等措施，缓解企业用工难。

（五）多措并举改善民生，社会事业全面进步。在连续三年省民生工程考核获奖的基础上，2011年又累计投入21.4亿元，全面完成33项民生工程。保障性住房建设工作位居全省前列。全市开工保障性住房10004套，基本建成5273套，竣工交付3443套，廉租住房实现保障12339户，全面超额完成省定目标任务。同时，还开工建设安置房近1.4万套，竣工8473套。创新建设模式，在普通商品房、拆迁安置房中配建公租房和廉租房。就业和社会保障工作继续加强。积极创建创业型城市，多渠道开发就业岗位，新增城镇就业近8万人，下岗失业人员再就业2万余人，登记失业率3.48%。超额完成五项社会保险扩面征缴任务，在全省率先实现城乡居民养老保险制度全覆盖。乡镇街道全部建立就业和社会保障事务所。城乡低保和农村五保供养标准得到提高。城乡医疗救助“一站式”服务平台开通运行。市社会福利中心开工建设。15所敬老院改扩建工程全部完工并投入使用。努力化解物价上涨压力。加强价格监管，建立市场价格调控工作联席会议制度，落实绿色通道等各项扶持政策，推进菜市场建设与改造、农超对接、公益性直销菜场试点工作。启动社会救助和保障标准与物价上涨联动机制，对五类低收入人群累计发放临时价格补贴3315万元，总量位居全省前列。加强“菜篮子”工程建设，市区新建大棚蔬菜基地1350亩，建成万头猪场2个，年出栏生猪5万头。加快发展文化教育体育事业。荣获“全国文化体制改革先进地区”称号。宣州、宁国、泾县荣获“全国科技进步先进县”称号。公共图书馆、博物馆和文化馆（站）全部免费开放。绩溪博物馆完成主体工程。建成39个乡镇综合文化站和67个广播电视“村村通”工程。提前一年实现行政村“村村有书屋”的目标。皖南花鼓戏《天下第一墨》被列为省重点文艺项目。承办了皖江八市第五届“新红歌”大赛。电视剧《文房四宝》拍摄项目顺利签约。完成68所义务教育阶段学校标准化建设，校安工程、留守儿童之家和校外活动室年度建设任务全部完成。制定学前教育三年行动计划，新改扩建幼儿园47所。高中阶段毛入学率达到90%。郎溪中学新校区建成使用。市职教中心被认定为国家级重点中等职业学校。宣城职业技术学院被列入省级重点支持建设院校。绩溪徽州学校挂牌成立。荣获“全国围棋之乡”称号。承办了省第六届农运会。举办了市首届中小学生运动会。不断提高医药卫生服务和人口计生工作水平。基层基本药物制度实现全覆盖。新农合门诊统筹总额预算管理全面启动。卫生应急网络化组织和管理体系基本形成，医疗质量监管网络已经建立。宣城中心医院、市精神病医院和骨科医院新建项目开工建设。食品药品安全监管进一步加强，广德、宁国、绩溪进入省级食品药品安全示范县。市残疾人康复中心项目扎实推进。各项人口指标继续位于全省前列，人口性别比持续保持正常，人口自然增长率控制在3‰以下。第六次全国人口普查登记工作顺利完成。全面推进文明创建工作和其它事业发展。新增郎溪新发镇、绩溪龙川村2个“全国文明村镇”，宣城中学、市供电公司2个“全国文明单位”，广德、宁国、绩溪荣获“省级文明城市”称号，46个单位成为“全省文明单位”。少数民族乡村经济发展不断加快。宗教事务依法管理得到加强。征兵任务圆满完成。工商联工作有了进一步加强和改进。外事侨务、新闻出版、档案、地方志、税务、防震减灾、气象等工作取得新进步。妇女、未成年人、老龄和红十字、慈善事业实现新发展。

（六）创新理念抓社会管理，社会局面和谐稳定。加强新形势下群众工作。扎实开展“四级书记大走访”和“千名干部下基层”活动，解决了一批群众反映的突出问题。深入开展网络“政民互动”在线访谈活动，认真办好“百姓热线”。组织开展“百案攻坚”，依法化解各类信访积案。建立健全了国有土地上房屋征收等项目社会稳定风险评估、被征地农民社会保障和就业培训等制度。加强社会综合治理。在荣获全国“五五普法先进集体”的基础上，全面启动实施“六五”普法规划。41个集体荣获“全国青少年普法教育先进单位”称号。深入推进平安宣城、法治宣城建设，加快建设大防控、大调解体系，完善矛盾纠纷排查调处机制，扩大社会治安视频监控系统覆盖面，7个县（市、区）全部成为省级平安县（市、区）。组织开展重点工程建设环境整治行动，有力保障了项目建设。加强社区建设和村务管理。圆满完成第八届村委会换届选举工作。扎实推进社区信息化建设试点。强化村务公开和民主管理，56个“难点村”治理任务全部完成，“阳光村务工程”通过省级达标验收。加强安全生产和应急管理。开展了打击违法生产经营建设行为、重点行业领域隐患排查整治行动和“清剿火患”战役，累计依法关闭煤矿、非煤矿山、危险化学品和烟花爆竹企业33家，安全生产态势平稳。打击侵犯知识产权和制售假冒伪劣商品专项行动取得明显成效。国防教育和双拥、优抚等工作得到加强。政府应急管理与国防动员、军队指挥体制机制衔接试点工作扎实推进，建成省内一流的人防（应急）指挥中心。

各位代表、各位同志，过去的一年，我们从严治政抓自身建设，政府的决策力、执行力和影响力得到进一步提高。一是探索完善工作机制。坚持和完善了规委会、编委会制度，实行了土地利用管理委员会、城市管理委员会集体议事制度，实施重点工程综合指挥调度机制，建立了重点项目、重点工程巡查推进制度，完善了部门牵头负责制和经济运行分析调度制度。进一步规范了土地征收、房屋拆迁、城乡规划、民生工程建设等领域重大决策行为。坚持市区目标、工作、利益、责任“四个一体化”，促进市区共同发展。二是巩固提升行政效能。继续深化民主考评活动，加大日常考评。强化行政审批效能督查，市、区行政审批事项统一进驻市政务服务中心办理，办结时限平均提速43.8%。三是不断加强廉政建设。开展了“以人为本、执政为民”和“加强作风建设、弘扬清风正气”学习教育活动。大力推进廉政风险防控。积极开展“政务公开推进年”活动。加快县级招投标中心达标建设，推进公共资源交易平台规范化。加强对转变发展方式和保障性安居工程、环境保护、重大建设项目等落实情况的监督检查，开展公共机构节能、机关公务用车、清理规范中介机构等专项治理工作。深化经济责任审计，强化政府投资审计，基本实现城市建设重点项目跟踪审计全覆盖。

各位代表、各位同志，过去的一年，全市上下凝心聚力谋发展、抢抓机遇促崛起，胜利实现“十二五”良好开局。这是省委、省政府和市委坚强领导的结果，是全市上下克难攻坚、团结拼搏的结果，也凝聚着全体人大代表、政协委员的智慧和力量。在此，我代表市人民政府，向全市广大工人、农民、知识分子、干部和老同志，向驻宣部队指战员、武警官兵和政法干警，向各民主党派和工商联、各人民团体和社会各界人士，致以崇高的敬意！向来我市创业的投资者、建设者，向所有关心支持宣城建设和发展的各界朋友，表示衷心的感谢!

我们也清醒地认识到，全市经济社会发展还存在很多问题和不足，主要体现在：一是经济总量小、企业体量小，大项目少、好项目少，城镇化水平低、产业层次低的“两小、两少、两低”状况尚未根本改变，转型发展仍需加快。二是融资难、用地难、征迁难和企业生产经营难比较突出，发展压力仍然较大。三是市本级发展仍需加快，中心城市带动能力亟待增强。四是工作推进难、群众办事难现象依然存在，政府服务效能仍需提高。对此，我们将采取更加有力的措施，切实加以解决。

二、2012年发展目标和重点工作

今年是实施“十二五”规划承上启下的重要一年。当前，我市正处在工业化、城镇化快速发展阶段和产业结构转型升级重要时期，多年积累的发展能量蓄势待发，内生动力不断增强，新的竞争优势正在形成，进一步发展的潜力巨大。但同时也面临着许多困难和挑战。我们既要在良好的势头中增强危机意识，也要在困难中坚定发展信心，始终保持奋发有为、积极进取的精神状态，把各种困难估计得更充分些，把应对之策谋划得更周全些，把各项工作推进得更扎实些，千方百计保持经济社会良好的发展势头。

今年政府工作的总体要求是：深入贯彻党的十七届六中全会、中央和全省经济工作会议、省第九次党代会及市第三次党代会的精神，坚持科学发展主题和转型发展主线，把握“稳中求进、进中求快、快中求好”这一重要原则，把保增长作为首要任务，把改革创新作为强大动力，把保障改善民生作为根本目的，为建设经济强市、文化强市、生态强市奠定更加坚实的基础。

全市经济社会发展的主要预期目标是：生产总值增长14%左右；规模工业增加值增长22%以上；财政收入增长20%以上；固定资产投资增长25%以上；社会消费品零售总额增长18%左右；外贸进出口总额增长25%以上，城乡居民收入增长15%以上。确保完成省定节能减排任务。

重点抓好以下八个方面工作：

（一）着力推进示范区建设，承接产业转移要有新突破。今年是实现示范区建设“三年见成效”阶段性目标的关键一年，必须坚持高端承接、绿色发展，推动示范区建设取得更大进展。加快园区扩容升级。积极推进市开发区争创国家级开发区工作。加快落实市开发区世行贷款项目，加强基础设施建设，进一步完善西扩区域配套功能。壮大汽车及零部件、新型建材、LED等主导产业，发展现代装备制造、新能源、节能环保、生物医药等新兴产业，市开发区财政收入要力争突破8亿元。继续支持宁国开发区创建国家级开发区。加强园区项目建设。确保市本级华明飞彩、星亚节能车业、纳察科技、鑫鸿汽车模具和宣酒工业园等项目建成投产，加快推进中鼎产业园、金达利液压设备和郎溪鸿泰特种钢、广德汽摩配产业园、泾县南华大电机、宁国凤形12万吨耐磨材料、绩溪高铁铝合金电缆、旌德金泉管业塑料管材等项目建设。增强招大引强力度。围绕战略性新兴产业、服务业、重大基础设施等方面，积极谋划一批大项目、好项目，争取国家和省更多的政策扶持。进一步改进招商方式，积极拓展境外招商，确保实际到位省外资金、实际利用外资分别增长25%以上。市开发区要力争引进2—3家投资超10亿乃至数十亿元的大企业、好项目。进一步加强与央企、知名民企的合作。加快郎溪经都产业基地等合作共建园区建设。

（二）着力加快转型升级步伐，壮大工业经济要有新进展。坚定不移走新型工业化道路，推动传统产业新型化、新兴产业规模化，加快构建现代产业体系。加速发展战略性新兴产业。统筹规划新兴产业发展基地，积极申报省级高新技术产业园，努力打造新兴产业集聚区。开工建设南漪湖风力发电、市区生活垃圾焚烧发电等项目。加快宣州博瑞特节能装备、郎溪特种设备、广德PCB、宁国医疗器械等产业园建设，推进宣州建林机械、郎溪动力源、广德金茂重工、宁国海成电子、泾县新能源汽车电机、绩溪抽水蓄能电站、旌德数控数显钻床等重点项目建设。增强自主创新能力。加速推进科技企业孵化器建设，完成市科技产业服务中心一期工程建设。争取新增“科技路路通”创新服务站2个、省级工程技术研究中心3家以上、省级企业技术中心5家以上，新增高新技术企业15家以上。确保广德通用汽车研发中心建成运营。深入推进产学研合作，积极开展技术创新，实施省级以上创新计划项目50个。力争全年高新技术产业产值达320亿元、增加值100亿元左右。实施质量品牌战略。扎实开展服务标准化试点、标准化良好行为企业和优质产品生产示范区等培育工作。组织开展全国知名品牌创建示范区申报工作。力争新创中国驰名商标2个和省名牌产品10个、著名商标38个。加快国家级文房四宝产品质量监督检验中心建设，确保宁国耐磨材料、泾县电机、绩溪不锈钢3个省级检验检测中心投入使用。改造提升传统产业。确保宣城海螺二期、宣城电厂二期等项目开工建设。推进广信农化光气化产品、黄山实业不锈钢链条等项目建设。实施中小企业促进工程，力争培育亿元工业企业200家以上。加强企业帮扶和运行调度。认真落实结构性减税等支持小微型企业发展政策，继续执行工业项目建设财政补助、企业融资财政贴息和规费减免、培训补贴等政策。强化金融支撑，加大对小微企业扶持力度，引导金融机构扩大信贷投放，力争全年新增贷款再过百亿。加强经济运行分析调度，进一步强化对企业的联系帮扶，加强融资、土地、用工等要素保障，帮助企业解决生产经营中的困难。鼓励支持企业开拓外贸市场，加强出口成长性企业的孵化培育，力争25家企业进出口过千万美元。强化资源和环境保护。坚持依法依规和节约集约用地，依法处置违法用地和闲置土地。加强矿产资源整合，推进高水平开发利用。继续淘汰落后产能，引导和鼓励企业使用高效节能设备。组织实施40个重点节能改造项目，突出抓好32户重点耗能企业的节能降耗工作。加快国家生态市和国家环保模范市创建步伐，支持郎溪、广德、泾县、旌德创建国家生态县。强化污染减排工作。规范污水处理厂运行管理，推进园区和乡镇污水处理设施和配套管网建设。提高城市生活垃圾无害化处理水平。加强规模化畜禽养殖企业污染治理和机动车尾气污染的监管。

（三）着力提升现代农业水平，新农村建设要有新举措。持续提升产业化水平。深入实施农业产业化“262”工程，加快现代农业示范区和农产品加工园区建设。稳定粮食生产。加快市粮食储备库建设，开工建设皖东南粮食批发市场。继续推进农产品精深加工，抓好立大禽业熟食加工和荣达禽业蛋品加工等项目建设，力争农产品加工产值超过400亿元。进一步推进农业标准化和品牌化，加强农产品购销平台和质量安全体系建设。发展壮大烟叶、茶叶、油茶、中药材等特色高效农业。深入实施畜牧业升级计划和竹林丰产增效、水产跨越和农机化推进三项工程。加强“菜篮子”基地建设，通过2至3年的努力，确保市区蔬菜基本实现自给。不断深化农村改革。认真开展宣州区农村房屋发证与抵押流转改革试点、郎溪县城乡一体化综合配套改革试点和宁国市统筹城乡土地使用制度综合改革试点。继续推进扩权强镇改革。积极稳妥推进土地承包经营权流转，加快农民专业合作社规范化建设，引导成立土地流转股份合作社。完善集体林权制度配套改革，推进林权抵押贷款和森林保险工作，加快林权管理信息和森林资产评估、收储、担保、交易平台建设。深入推进美丽和谐乡村建设。继续实施“百村十镇示范工程”，深入推进全市新农村10条示范带建设，新增50个美丽和谐村庄，继续打造10个文化特色村。加强村镇规划、建房管理和农村危房改造工作。继续开展农村环境综合整治，实施农村清洁工程。加强村级财务管理，继续推进“阳光村务”。

（四）着力强化载体建设，服务业发展要有新成效。把推动服务业大发展作为全市产业转型升级的战略重点，促进服务业发展提速、比重提高、水平提升。培育壮大商贸物流业。加快物流园区基础设施建设。推进中国茶府、百汇商贸和农副、农资产品批发市场等项目建设，纺织服装批发市场、联鑫家电市场、农机市场、国际竹艺商贸城、冷链物流、亚夏汽车文化财富广场等项目都要开工建设。加强城乡流通体系建设，培育壮大商贸流通企业，力争新增限额以上企业100家。加快国购广场、星隆国际广场建设。开工建设市区大润发超市。加强城区菜市场标准化建设。开展“万村千乡市场工程”信息化改造试点。加快“新网工程”和再生资源回收利用交易市场建设。积极申报全国再生资源回收利用体系试点城市。大力推进电子商务。支持宣州区申报全省商贸服务业发展示范区。大力发展金融业。力争引进招商银行、民生银行等金融机构，继续组建新的村镇银行，做好小贷公司、融资性担保公司监管工作。引导民间合法借贷，及时处置和打击非法集资行为，清理整顿非融资性担保机构。深入推进金融产品创新，完善社会信用体系建设，优化金融生态环境。积极引进风险投资，培育上市后备资源，抓好广信农化、黄山胶囊、五星食品、凤形耐磨、聚隆集团等企业上市工作。做大做强旅游业。进一步整合旅游资源，筹建市旅游发展投资公司。进一步完善敬亭山基础设施，启动江南诗山文化园项目。开展龙川、大云岭红色景区5A创建和龙川申报国家级风景名胜区工作。加快扬子鳄自然保护区、广德和合生态园、恩龙世界木屋村总体提升工程、旌德黄山东线生态旅游走廊等项目建设。大力发展乡村旅游。启动南漪湖总部经济园区和生态旅游度假区项目。加强宣传推介，推出一批旅游精品线路。加大文房四宝等特色旅游商品的研发。加快敬亭湖宾馆、万豪酒店等建设进度。同时，建立健全房地产市场体系和合理的住房供应体系，推进房地产业健康规范发展。

（五）着力加强基础设施建设，城镇化水平要有新提升。坚定不移地推进城市、交通、水利等建设，充分发挥基础设施对经济社会发展的支撑和保障作用。市本级安排城市规划建设项目176个，总投资近400亿元，年内完成投资171亿元。加快实施城市建设“四大工程”。提高规划水平，强化规划引导作用，加强规划执行的监管。一是畅通性工程建设。继续推进城区“二环八射”路网建设。重点建设水阳江大道闭合段和宝城路改造。续建和新建环城大道等41条道路和28个市政配套项目。做好环城大道8标段、阳德路改造等9个项目前期工作。二是功能性工程建设。力争合工大宣城校区一期投入使用。完成宣中新校区主体工程。推进市体育中心、图书馆、规划馆和海关、出入境检验检疫局项目建设。做好市博物馆新馆、宣城大剧院、科技馆、美术馆、档案馆新馆等项目前期工作。完成仁和时代广场建设。启动彩金湖政务新区项目。三是休闲性工程建设。继续推进“一山一河一湖多园”工程。建成敬亭湖公园、宣广高速宣城东出口公园。完成扬子鳄湖（暂定名）西湖景区主体工程，开工建设东湖景区及宛溪河综合整治二期工程。加快新港生态湖建设。整治泥河、道叉河。四是保障性工程建设。积极推进北门等棚户区改造，加快建设夏渡新城、桂花园、彩虹小区、付山新村等12个保障性住房和拆迁安置区工程。有序开展石油供应网点和城市燃气管网建设。同时，注重城管规范化、长效化和精细化，完善“数字城管”平台。健全禁违查违拆违联动机制，努力遏制违法建设。大力推进县城和中心镇建设。加快综合交通体系建设。完成芜雁高速和宣广高速宣城东、西出入口改扩建工程。加快宣宁、宁绩、宁千高速建设。力争开工建设溧广高速。做好广宁、绩溪至谭家桥高速和鸿门至黄山连接线等项目前期工作。完成老318祠界路改建并通车，完成宣狸快速通道、梅岭隧道连接线、绩溪至长安段改建等主体工程。加快推进205国道宣城段示范工程、宣泾快速通道建设和214省道郎溪段改建、215省道绩溪至歙县段改造工程。认真开展广德至宜兴段、蔡家桥至土桥段、旌德至长安段改造和水阳江航道整治工程前期工作。完成芜申运河宣州段建设，加快定埠段建设。力争开工建设市汽车客运总站。加强农村公路、危桥改造等工程建设。加快推进城乡客运一体化进程。全力配合推进合福高铁宣城段建设，积极配合做好商杭高铁、皖赣铁路扩能改造等项目前期工作。加快水利设施建设。强力推进水阳江下游“开卡”工程建设。继续加快城东联圩和水库除险加固工作。启动双桥联圩排涝泵站和堤防应急加固除险工程。推进青弋江灌区续建配套、港口湾灌区和郎源水库扩容、汤村水库、粮长门水库等工程前期工作，启动水阳江中游、新老郎川河综合治理等项目前期工作。继续推进农村安全饮水工程建设。加强水利工程的建后管护工作。加快电力、通信设施建设。认真做好电网“十二五”规划滚动调整和对接工作，启动市电力专项规划修编工作。建成投产110千伏市中心变、220千伏桃州变扩建等5项输变电工程，加快推进220千伏太平、110千伏金河等7项输变电工程建设。加快特高压主干电网建设和农网改造升级。加大通信设施的建设与保护，推动以3G为代表的现代通信技术发展和信息化产品运用，促进“无线城市”建设。

（六）着力发展文化产业，文化发展繁荣要有新气象。以更大力度推进文化改革和发展，加快实现由文化资源大市向文化产业强市跨越。坚持以项目带动文化产业的发展。力争跻身省级文化产业示范区。完善扶持政策，积极引进文化产业项目。加快建设夏渡文化创意产业园区，推进中国书画交易中心、中国工艺礼品城等项目建设，力争徽文化展示基地、动漫衍生品交易基地和少儿科普产业基地等项目开工建设。积极谋划广告传媒产业基地项目。坚持以工程推动文化事业的繁荣。继续实施文化惠民工程，加快重点公共文化设施建设。加强文化遗产和文物的保护与开发。启动广德太极洞国家文化和自然遗产保护设施建设项目，实施绩溪历史文化街区保护设施建设项目。推进谢朓楼重建工程。加强文艺精品创作，积极做好电视剧《文房四宝》拍摄工作。坚持以改革激发文化活力的增强。培育新型文化市场主体，加快宣城报业集团、广电集团和市文化产业投资公司组建工作。推进市广电文化产业中心建设。支持中国宣纸集团、皖南海峰印刷包装公司等企业做大做强。

（七）着力保障改善民生，社会事业要有新发展。切实办好涉及民生的大事实事和好事难事，让人民群众更多更快更好地享受发展成果。扎实推进民生工程。继续加大投入，确保完成省定33项民生工程建设任务。落实和完善应对价格异动应急预案和工作机制，设立价格调节基金，加强民生商品价格监管。努力提高居民收入。全面实施居民收入倍增规划。实施创业富民工程，深化省级“创业型城市”创建活动。建立健全职工工资正常增长和支付保障机制。推行企业工资集体协商制度，逐步提高最低工资标准。积极发展农村非农产业，扩大农村产权制度改革试点，构建农民增收多元支撑体系。落实和完善强农惠农政策，加强农民负担监管。扎实做好新阶段扶贫开发工作。继续加强就业和社会保障工作。实施更加积极的就业政策，加强就业培训，重点抓好城镇就业困难人员、高校毕业生和农民工就业，大力促进农村劳动力就近就地就业。加快建立统一规范的人力资源市场，加强人才引进和使用。扎实做好社会保险扩面提标工作，进一步完善城乡居民养老保险制度，促进社会保障体系更加健全，基金运行更加安全有效。提高城乡低保水平。完善城乡医疗救助“一站式”平台建设。确保市社会福利中心建成使用。各县（市）至少新建或改扩建1所社会（儿童）福利中心。支持社会化护理院建设。加强农村敬老院建设，提高“五保”集中供养率。加快市残疾人康复中心建设。加快发展教育体育人口卫生等事业。加速推进学校布局规划和标准化建设，促进学前教育和义务教育均衡发展。力争新改扩建幼儿园40所，学前三年适龄幼儿毛入园率达到65%。加快发展职业教育，推进旌德和众高等职业教育产业园一期工程和宣城工业学校二期项目。继续深入开展全民健身活动。开展县级公立医院改革试点。深化卫生服务管理体制和运行机制改革，加强农村和社区卫生服务体系建设。完善大中专毕业生到乡村卫生院（室）工作的激励政策。健全重大疾病防控和公共卫生应急体系，提高医疗质量和服务水平。加快宣城中心医院、市精神病医院等项目建设。深化全国人口和计划生育综合改革示范市建设，确保各项指标继续保持全省领先水平。制定并实施好新一轮妇女儿童发展规划，提高妇女儿童健康水平。加强统计基层基础工作。推进老龄事业和红十字、慈善事业健康发展。做好民族宗教、外事侨务、对台、档案、地方志、防震减灾、气象、烟草专卖等各项工作。

（八）着力加强社会管理创新，综合治理工作要有新作为。完善党委领导、政府负责、社会协同、公众参与的社会管理格局，加强体制机制的创新，提高综合治理水平。建立健全矛盾纠纷调处体系。落实重大事项社会稳定风险评估机制，从政策层面防止和解决社会矛盾。健全群众工作网络，加强人民调解、行政调解、司法调解工作，组建市仲裁委员会，加强医疗纠纷、道路交通事故等专业性第三方调解组织建设。严格依法处置“两堵两闹”等违法行为。推进劳动保障监察平台建设，切实做好农民工工资支付保障工作，加强劳动安全管理和职业病防治。加快群众来访联合接待服务中心和群众服务平台建设，继续开展领导干部开门接访等活动和积案化解工作，努力解决群众反映强烈的问题。建立健全社会事务管理体系。完善社区建设投入保障机制，加强社区建设，提高社区管理和服务水平。以乡镇、街道综治办和综治工作中心规范化建设为重点，加强综治基层基础建设。完善“四级联动”的工作体制，加快推进流动人口基本公共服务均等化。积极开展帮扶救助关爱行动和安置帮教、社区矫正工作，加强青少年及重点群体管理。关心留守儿童和空巢老人。积极创建司法所规范化建设示范县（市、区）。扩大法律援助覆盖面。强化网络舆情监控，加强对突发事件和群体性事件的网上舆论引导。发挥工会、共青团、妇联、红十字会等人民团体作用，支持其参与社会管理和公共服务。建立健全社会公共安全体系。完善综合防控体系，继续开展打黑除恶专项行动，严厉打击各类犯罪行为。严格落实安全生产责任制，强化重点行业、重点领域和重点部位的安全监管，加强隐患排查治理，确保安全生产形势持续稳定。以铁的手腕，辅之以一定的经济奖励，上半年要彻底关闭全市所有煤矿。切实加强学校及周边治安环境整治，认真做好中小学生和幼儿交通安全工作。加强食品药品安全日常监管和专项整治。坚持军民融合式发展，巩固深化“三个机制”衔接试点成果，整合应急资源和力量，增强应对突发事件能力。大力支持驻宣部队建设，加强国防教育、国防动员和双拥共建工作。

三、切实加强政府自身建设

面对发展的新形势、新难题、新要求，政府工作必须坚持做到“四个一”：

坚持“一盘棋”思想。实践证明，任何一个地方的发展，都是一个系统工程，其中任何一个环节都不能“停车断档”，否则就会事倍功半，甚至一事无成。全市各级政府及政府各部门必须坚持“守土有责”，多做、早做、做好各自份内的事。特别要自觉维护市委权威，自觉接受市人大、市政协监督，自觉接受各民主党派和工商联的监督，自觉接受群众和舆论监督，真正做到无条件服从服务于宣城的大发展，形成肝胆相照、荣辱与共，齐心协力、干事创业的生动局面。

坚持“一抓到底”。做任何工作，做过了不等于落实，只有做好了才是落实。要坚持算着干、现场看、盯着办，具体抓、抓具体。将工作重心、工作精力聚焦到项目上，并实行“挂图式作战”、“一线工作法”、重大项目督查巡查等工作机制，强化绩效管理考核、政务督查和行政问责，把工作盯紧抓实，切实防止工作在层层部署、层层要求中“信号”衰弱甚至中断的问题。只有把“信号”传到位，把工作抓到底，这样的落实才可谓“功夫到家”。

坚持“一刻不放松”。抓发展必须抢机遇，抢机遇必须只争朝夕。我们要求发展环境要更加宽松，但环境宽松不等于工作放松，工作放松就等于自我放弃。必须以“慢不得”的危机感、“等不起”的紧迫感、“坐不住”的责任感抓工作，树立雷厉风行、说干就干的作风，认真做好每件事，绝不放松每一天。对确定的事情快办办好、不推不拖不糊，对没有完成的工作要“忍不住、放不下”，再细化工作目标，量化进度指标，倒排工作计划，及时组织实施。

坚持“一身正气”。一个人有正气，才能赢得别人尊重；一个干部有正气，才能受到群众拥护。要认真落实党风廉政建设责任制，全面推进廉政风险防控，建立重点项目廉政报告备案等制度，强化审计监督，深化民主考评活动，深入开展工程建设等领域突出问题专项治理，严肃查处违法违纪案件，严肃查处损害群众利益、破坏发展环境的行为。同时，要把事业看重，把得失看轻，把名利看淡，真正做到胸怀坦荡做人、公道正派处事、勤政廉政为民。

各位代表、各位同志，今天的宣城，发展成就令人鼓舞，美好未来催人奋进。让我们在中共宣城市委的坚强领导下，全面落实科学发展观，在抓发展上求突破、勇争先，在惠民生上做好事、办实事，在促和谐上见分晓、比高低，同心同德、奋力拼搏，为建设经济繁荣、生态良好、社会和谐、人民幸福的美好宣城而努力奋斗！
